# Applying sequential pattern mining to investigate cerebrovascular health outpatients’ re-visit patterns

**DOI:** 10.7717/peerj.5183

**Published:** 2018-07-09

**Authors:** Chao Ou-Yang, Chandrawati Putri Wulandari, Rizka Aisha Rahmi Hariadi, Han-Cheng Wang, Chiehfeng Chen

**Affiliations:** 1 Department of Industrial Management, National Taiwan University of Science and Technology, Taipei, Taiwan; 2 Department of Information System, Universitas Brawijaya, Malang, Indonesia; 3 Department of Industrial Engineering and Management, Bandung Institute of Technology, Bandung, Indonesia; 4 Department of Neurology, Shin Kong Wu Ho-Su Memorial Hospital, Taipei, Taiwan; 5 College of Medicine, National Taiwan University, Taipei, Taiwan; 6 College of Medicine, Taipei Medical University, Taipei, Taiwan; 7 Department of Public Health, School of Medicine, College of Medicine, Taipei Medical University, Taipei, Taiwan; 8 Division of Plastic Surgery, Department of Surgery, Wan Fang Hospital, Taipei Medical University, Taipei, Taiwan; 9 Cochrane Taiwan, Taipei Medical University, Taipei, Taiwan

**Keywords:** Sequential Pattern Mining, Cerebrovascular disease, Re-visit behavior patterns.

## Abstract

**Background and Objective:**

Increases in outpatients seeking medical check-ups are expanding the number of health examination data records, which can be utilized for medical strategic planning and other purposes. However, because hospital visits by outpatients seeking medical check-ups are unpredictable, those patients often cannot receive optimal service due to limited facilities of hospitals. To resolve this problem, this study attempted to predict re-visit patterns of outpatients.

**Method:**

Two-phase sequential pattern mining (SPM) and an association mining method were chosen to predict patient returns using sequential data. The data were grouped according to the outpatients’ personal information and evaluated by a discriminant analysis to check the significance of the grouping. Furthermore, SPM was employed to generate frequency patterns from each group and extract a general association pattern of return.

**Results:**

Results of sequence patterns and association mining in this study provided valuable insights in terms of outpatients’ re-visit behaviors for regular medical check-ups. *Cosine* and *Jaccard* are two symmetric measures which were used in this study to indicate the degree of association between two variables. For instance, *Jaccard* values of variable abnormal blood pressure associated with an abnormal body-mass index (BMI) and/or abnormal blood sugar were respectively 47.5% and 100%, for the two-visit and three-visit behavior patterns. These results indicated that the corresponding pair of variables was more reliable when covering the three-visit behavior pattern than the two-visit behavior. Thus, appropriate preventive measures or suggestions for other medical treatments can be prepared for outpatients that have this pattern on their third visit. The higher degree of association implies that the corresponding behavior pattern might influence outpatients’ intentions to regularly seek medical check-ups concerning the risk of stroke. Furthermore, a radiology diagnosis (i.e., magnetic resonance imaging or neck vascular ultrasound) plays an important role in the association with a re-visit behavior pattern with respective 50% and 70% *Cosine* and *Jaccard* values in general behavior {f11}∧{f01}. These findings can serve as valuable information to increase the quality of medical services and marketing, by suggesting appropriate treatment for the subsequent visit after learning the behavior patterns.

**Conclusions:**

The proposed method can provide valuable information related to outpatients’ re-visit behavior patterns based on hidden knowledge generated from sequential patterns and association mining results. For marketing purposes, medical practitioners can take behavior patterns studied in this paper into account to raise patients’ awareness of several possible medical conditions that might arise on subsequent visits and encourage them to take preventive measures or suggest other medical treatments.

## Introduction

Increased public awareness of health conditions is encouraging people to seek medical check-ups, which has led to a significant growth in the number of medical data records. Medical check-ups serve several functions, such as evaluating one’s health status, screening for risk factors of potential diseases, following-up on treatment of a disease, and educating people to avoid certain environmental exposures that may have hidden risks of diseases (Chiu & Yeh, 2010; Hill, Frappier-Davignon & Morrison, 1979). Such medical services and treatments are provided to outpatients according to their needs. However, if the numbers of outpatients visiting hospitals for medical check-ups are unpredictable, hospitals may be unable to provide the required resources and facilities (Mestre, Oliveira & Barbosa-Póvoa, 2015). The limited facilities and resources may also affect the quality of the medical services offered by a hospital (Ma & Demeulemeester, 2013). Therefore, to prevent such conditions, strategic planning is necessary to investigate outpatients’ re-visit behavior patterns. According to Williams & Fitton (1988), the initial condition of an outpatient may affect his/her decision on whether to return for a regular medical check-up to follow-up on the results of a previous medical check-up.

A better understanding of outpatients’ re-visit behavior patterns is expected to allow medical practitioners to take the behavior patterns studied in this paper into account to increase outpatients’ awareness of several possible medical conditions that might arise on subsequent visits and encourage them to take preventive measures or to suggest other medical treatments offered by physicians. The offered service could be either regular medication for hypertension, diabetes, and/or cholesterol problems or any special treatment related to the risk of stroke in accordance with outpatients’ re-visit behavior patterns that have been discerned. The more details medical practitioners know about outpatients’ re-visit behavior patterns, the better they can increase outpatients’ awareness about taking preventive measures or suggest other medical treatments and encourage them to undergo regular medical check-ups.

As the importance of health examinations has attracted increased attention, much research related to health examinations has been conducted. However, few studies have attempted to predict the re-visit patterns of outpatients for medical check-ups. In general medicine, many studies have discussed ways to predict the demands of patients or outpatients. For instance, McCarthy et al. (2008) discussed predicting demand in emergency departments, while Al Nuaimi (2014) addressed predicting demand in healthcare services, and Batal et al. (2001) discussed predictions of patient visits at urgent care clinics. Those studies noted that predicting demand is an important aspect of strategic planning. Considering such conditions, outpatients’ return visits can be predicted by utilizing their health examination records, and data mining techniques can be employed to mine useful information from those data (Liao, Chu & Hsiao, 2012).

Data mining in the medical field has special features, such as its heterogeneity, ethical considerations, statistical features, and medicine’s special status. Therefore, data mining in the medical field has become a special research topic, such as the comprehensive reading of health medical check-up reports and predicting diseases from them (Koh & Tan, 2005; Ohara et al., 2013; Suka et al., 2014). However, to the best of our knowledge, few studies have tried to predict patterns of subsequent hospital visits for medical check-ups by considering association patterns based on outpatients’ medical data. In previous research (Kontio et al., 2014), machine learning techniques were used to predict patient acuity (a patient’s care needs), sequential pattern mining (SPM) was used for predicting some types of diseases (Ohara et al., 2013), and association rule mining (ARM) for physician prescriptions based on the history of outpatients’ re-hospitalization was performed by Ou-Yang, Agustianty & Wang (2013). In the medical field, SPM is commonly used to identify frequent patterns or behaviors that appear in datasets. Regarding previous research, both SPM and ARM are considered suitable methods, because the former uses sequential data as the input, commonly for prediction purposes, while the latter can be used to generate associations among features as references to help predict re-hospitalizations. Combining both SPM and ARM methods can facilitate the identification of frequent patterns and the development of general pairs of variables in medical records associated with a patient’s return.

This study used a cerebrovascular health examination dataset from a local medical center in Taiwan to identify outpatients’ re-visit behavior patterns for regular medical check-ups. The data were first grouped by outpatients’ personal variables (i.e., age and medical history of diabetes or hypertension), and each group was evaluated by a discriminant analysis to check the significance of the grouping results. Furthermore, frequent sequence patterns of health report variables (e.g., blood pressure, body-mass index (BMI), and blood sugar) were extracted by SPM. According to the extracted frequency sequence pattern from the previous step, some pairs of health variables with high influence on patient return were generated using the ARM technique. Results of this study can be used as a reference by hospitals to identify patterns that will encourage outpatients to return in the future for medical check-ups or consultations.

## Related Work

### Sequential pattern mining (SPM)

SPM is a method to discover all sequential patterns with user-specified minimum support. The support value of a pattern is the number of data sequences that contain the pattern (Agrawal & Srikant, 1995). The SPM method is commonly used to identify frequent patterns or behaviors that appear in a dataset. In the medical area, some methods have been proposed. For instance, the Time-Annotated Sequence algorithm was used to assess the effectiveness of extracorporeal photopheresis (ECP) from a liver transplant dataset (Berlingerio et al., 2007), and Ali et al. (2008) mined routine behaviors in a pervasive healthcare environment using the Routine Tree approach.

### Association rule mining (ARM)

ARM, a task that discovers hidden correlations among items in a large database (Wang & Shao, 2004), has received considerable attention, particularly since the publication of the AIS and Apriori algorithm (Agrawal, Imielinski & Swami, 1993; Agrawal & Srikant, 1994). In the medical area, it has been applied to identify relationships among specific drugs or specific diseases. For instance, Soni et al. (2011) used association mining to predict or diagnose a heart attack by seeing the correlation between some attributes associated with the heart attack. Ji et al. (2011) discussed implementing association mining for the early detection of unknown adverse drug reactions (ADRs). In Taiwan, the National Health Insurance Research Database managed by the Taiwan National Health Insurance has implemented association mining in several studies, for instance analyzing the co-prescribing pattern of antacid drugs (Chen, Liu & Hwang, 2002), exploring the comorbidity of attention deficit/hyperactivity disorder (ADHD) (Tai & Chiu, 2009), and investigating pairs of drugs associated with Stevens-Johnson syndrome (SJS) re-hospitalization (Ou-Yang, Agustianty & Wang, 2013).

In general, several studies have shown the importance of predicting demand in medical practice due to its usefulness for strategic planning. For instance, predicting demand in an emergency department (McCarthy et al., 2008) and healthcare services (Al Nuaimi, 2014), and predicting patients’ visits at an urgent care clinic (Batal et al., 2001) were discussed. However, to the best of our knowledge, only a few studies have investigated characteristics of re-visit patterns of patients, especially using a cerebrovascular health examination dataset. Therefore, this study combined some methods used by previous research to delve into the issue. The proposed methodology consists of three main parts: data discretization, SPM, and association mining.

The data discretization portion of our proposed method employs equal-frequency discretization (EFD), adapted from Wong & Chiu (1987) and Garcia et al. (2013). The main part of our methodology is a priori-based sequential pattern mining first proposed by Agrawal & Srikant (1995). This method is used to detect useful information of frequent (totally or partially ordered) subsequences, and is commonly used in marketing to identifying customer behaviors. This study adapted this method for identifying outpatients’ re-visit behaviors towards regular medical check-up activity, in terms of a risk of stroke. The last portion of our proposed method adopted a priori-based association mining, which was used to investigate pairs of drug associated with SJS re-hospitalization (Ou-Yang, Agustianty & Wang, 2013). In our study, this method was used to investigate pairs of health factor variables associated with outpatients’ re-visit behaviors. Some adjustment needs to be considered to combine the association mining method with pattern mining in the previous step.

**Figure 1:**
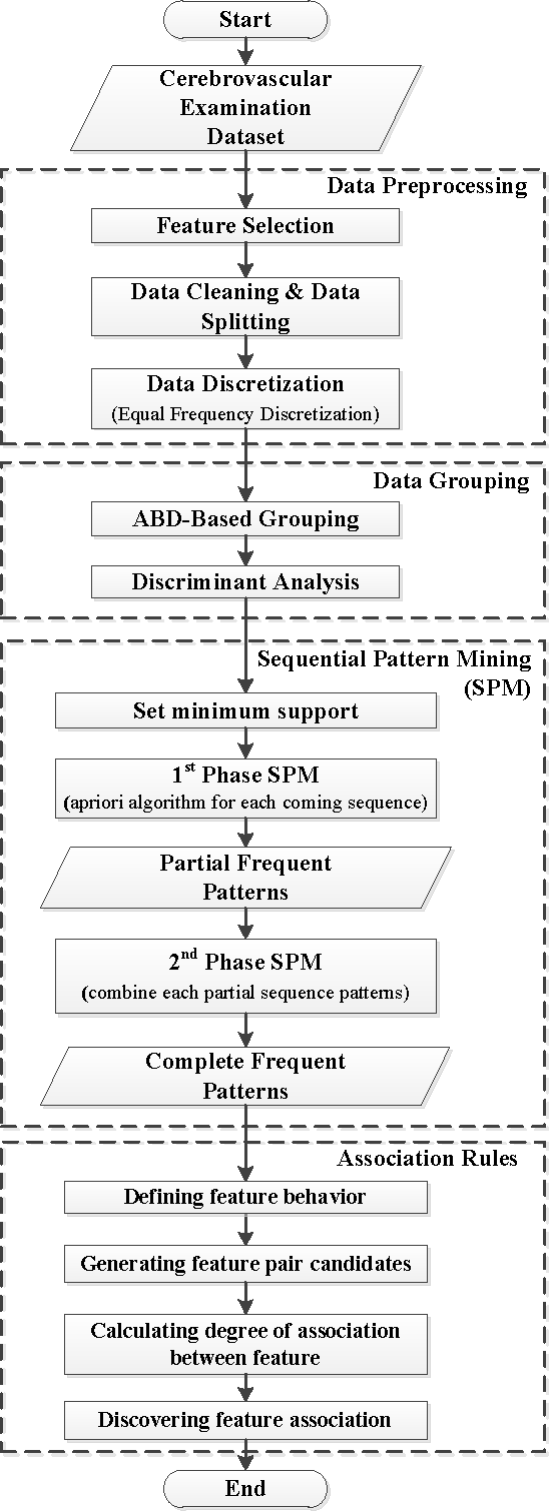
Research framework.

## Methods

Figure 1 presents the general framework, which contains the four main parts of the proposed method: data preprocessing, data grouping, SPM, and ARM.

### Data collection and data preprocessing

A seven-year (2004–2011) collection of cerebrovascular health examination data records from a local medical center in Taiwan was used in this study. It consisted of 13,692 medical check-up records from 12,062 outpatients. A detailed description of the dataset is presented in Table 1.

In the beginning of the process, data preprocessing was applied to remove noise from the data, extract and merge data from different sources, and convert the data into a proper format (Hu, 2003). The procedures to conduct data preprocessing are described as follows.

#### Step 1. Feature selection

In this step, commonly used variables related to patient return cases were chosen based on stroke risk factors of age, blood pressure history, and diabetes history (ABD) (Reckless & Buchan, 2008). As a result, further analysis of this research was related to ABD and seven other health factor variables that most often appeared in previous research on medical check-ups, such as blood pressure (BP), the BMI, neck vascular ultrasound (NV), magnetic resonance imaging (MRI) of the brain (MB), blood sugar (BS), cholesterol (CH), and triglycerides (T). After selecting those variables based on a literature review, experts were consulted for validation to ensure that these variables were correctly chosen.

#### Step 2. Data cleaning

Data records containing missing values, illogical data, or unused data values were removed. In accordance with the original data, 748 data records from 543 examinees were removed representing respective percentages of 5.46% and 4.5%.

#### Step 3. Data splitting

In this step, the dataset was split into groups of visits based on outpatients’ frequency of visits (*c-* visits), indexed by *c* = 1, 2, …, C, where *C* is the maximum value of the frequency of visits in the dataset. Because the first visit was an initial medical check-up, it can be assumed that the patient’s frequency of visits is (*c –1*). This study only focused on outpatients whose medical data records contained two to four visits to get a return reference sequence pattern for outpatients on their first to third visits.

**Table 1:** Detailed description of the dataset.

No. of visits	No. of outpatients	Percentage	Time interval among visits (approx.)
1 visit	10,404	86.25%	–
2 visits	885	7.34%	3∼5 years
3 visits	165	1.37%	1∼2 years
4 visits	49	0.41%	1∼2 years
Others	559	4.63%	0.5∼1 year

#### Step 4. Data discretization

Discretization aims to transform a set of continuous variables into a discrete form in order to reduce the total number of pattern combinations in the SPM method (Kotsiantis, Kanellopoulos & Pintelas, 2006). Discretization was performed on a set of continuous variables, which contains five health factor variables (BP, BMI, BS, CH, and T). Those continuous variables were derived in accordance with National Institute of Health standards and were classified into two general categories (normal and abnormal) as shown in Table 2. The personal variable, age, was discretized by the equal-frequency discretization (EFD) method. The EFD method is considered suitable for the characteristics of our problem, which are static, univariate, and unsupervised (Garcia et al., 2013; Liu et al., 2002; Wong & Chiu, 1987). A performance evaluation was conducted by means of the standard deviation of the frequency because it can handle imbalanced data from each group. As a result, the EFD method can discretize the variable age into four categories with the lowest standard deviation value.

### Data grouping of ABD variables

#### Initial group characteristics of ABD variables

Different backgrounds of personal data variables can affect different risk levels of stroke, so the dataset was grouped in accordance with combined ABD variables (Reckless & Buchan, 2008). In general, if there are *r* variables, the *i*th of which contains *n*_*i*_ representative categories, then the total number of possible combinations of ABD variables (*N*) can be computed using Eq. (1):

(1) \begin{eqnarray*}N=\prod _{i=1}^{r}{n}_{i}.\end{eqnarray*}

***Example:***

For the dataset used in this study, ABD consists of three variables (*r* = 3). Let *i* be the index of ABD variables, in which *i* = 1, 2, …, *r* denotes the personal variables age, blood pressure history, and diabetes history, respectively. Each personal variable contains a number of representative categories, *n*_*i*_, for the respective ABD variables. Table 2 shows that the variable age has four categories (*n*_1_ = 4), and there are three categories each for hypertension and diabetes history (*n*_2_ = *n*_3_ = 3). Hence, the total number of groups of combinations of ABD variables is 36.

Neither machine learning nor a statistical model is required to obtain the characteristics of the combined variables in our work because the rule for basic combination in accordance with the data characteristics is already clear. Furthermore, note that the initial combinations tabulated in Table S1 are group characteristics of ABD variable combinations before considering changes in group characteristics due to the time interval among visits (Table  S2).

**Table 2:** Discretization results.

No	Variable	Range	Category	Source
1	Blood Pressure (BP in mmHg)	60∼79 (diastole); 90∼119 (systole)	Normal (BPN)	National Institutes of Health’s Standard
		<60 or >79 (diastole) <90 or >119 (systole)	Abnormal (BPA)	
2	Body Mass Index (BMI in kg/m^2^)	18.5∼24.9	Normal (BMIN)	
		<18.5 or >24.9	Abnormal (BMIA)	
3	Neck Vascular Ultrasound (NV)		Normal (NVN) Abnormal (NVA)	
4	MRI brain (MB)		Normal (MBN) Abnormal (MBA)	
5	Blood Sugar (BS in mg/dL)	<110	Normal (BSN)	
		≥110	Abnormal (BSA)	
6	Cholesterol (CH in mg/dL)	<200	Normal (CHN)	
		≥200	Abnormal (CHA)	
7	Triglyceride (T in mg/dL)	<150	Normal (TN)	
		≥150	Abnormal (TA)	
8	Age (years)	26∼46 47∼52 53∼58 59∼87	Age1 Age2 Age3 Age4	EFD Method
9	Hypertension History		Yes, No, Unchecked	
10	Diabetes History		Yes, No, Unchecked	

#### Identifying additional groups of possible changes in characteristics

Due to the time interval of clinical visit activity, it is natural that characteristics of outpatients’ personal variables could change at every clinical visit in terms of age. Therefore, some additional groups are required to represent those characteristic changes, especially for the three-visit and four-visit datasets. For instance, suppose an outpatient had their first clinical visit at Age1 (age range 26–46 years), with a history of blood pressure (hypertension) and a history of diabetes; then his/her personal characteristic changes into Age2 (age range 47–52 years) at the second visit. This means that in accordance with the initial combinations of groups presented in Table S1, the outpatient’s personal characteristic at the initial visit is g-1, which then changes to g-10 at the second visit. Therefore, this changing condition is presented as group III-37. The identification process is continuously performed until no more changes in characteristics are found. The same procedure is performed on the four-visit dataset, and the final results of additional group characteristic for the three-visit and four-visit datasets are presented in Table S2. As a result, Table S2 shows 15 additional groups for the three-visit dataset and 20 additional groups for the four-visit dataset.

After considering all possible characteristic changes and identifying all additional groups, the initial grouping result of ABD variables is updated, as is the information about the total number of groups with cardinality of <10. This result is presented in Table S3.

#### Discriminant analysis

According to Hair et al. (2006), a discriminant analysis is applicable to any research with the objective of understanding group membership. Our proposed method grouped variable characteristics in which each group satisfies the predefined cardinality threshold. Otherwise, the group needs to be merged with another group which is closest in distance. The distance between centroids of the groups is computed by means of the Euclidean distance. A discriminant analysis was conducted using SPSS software to check significant differences between groups of visits with respect to health variables.

In addition, Box’s M test is a statistical descriptive test in the discriminant analysis to test the equality of covariance matrices. In terms of the equality of variance–covariance matrixes, it can be assumed and concluded that the grouping result is good if the statistical significance value does not exceed a critical level (5%) (Hair et al., 2006). Otherwise, alternative statistical methods should be considered to evaluate the equality of variance–covariance matrices. The detailed experimental protocol for conducting group merging and the discriminant analysis is described in Table S4.

### Mining sequential patterns

**Figure 2:**
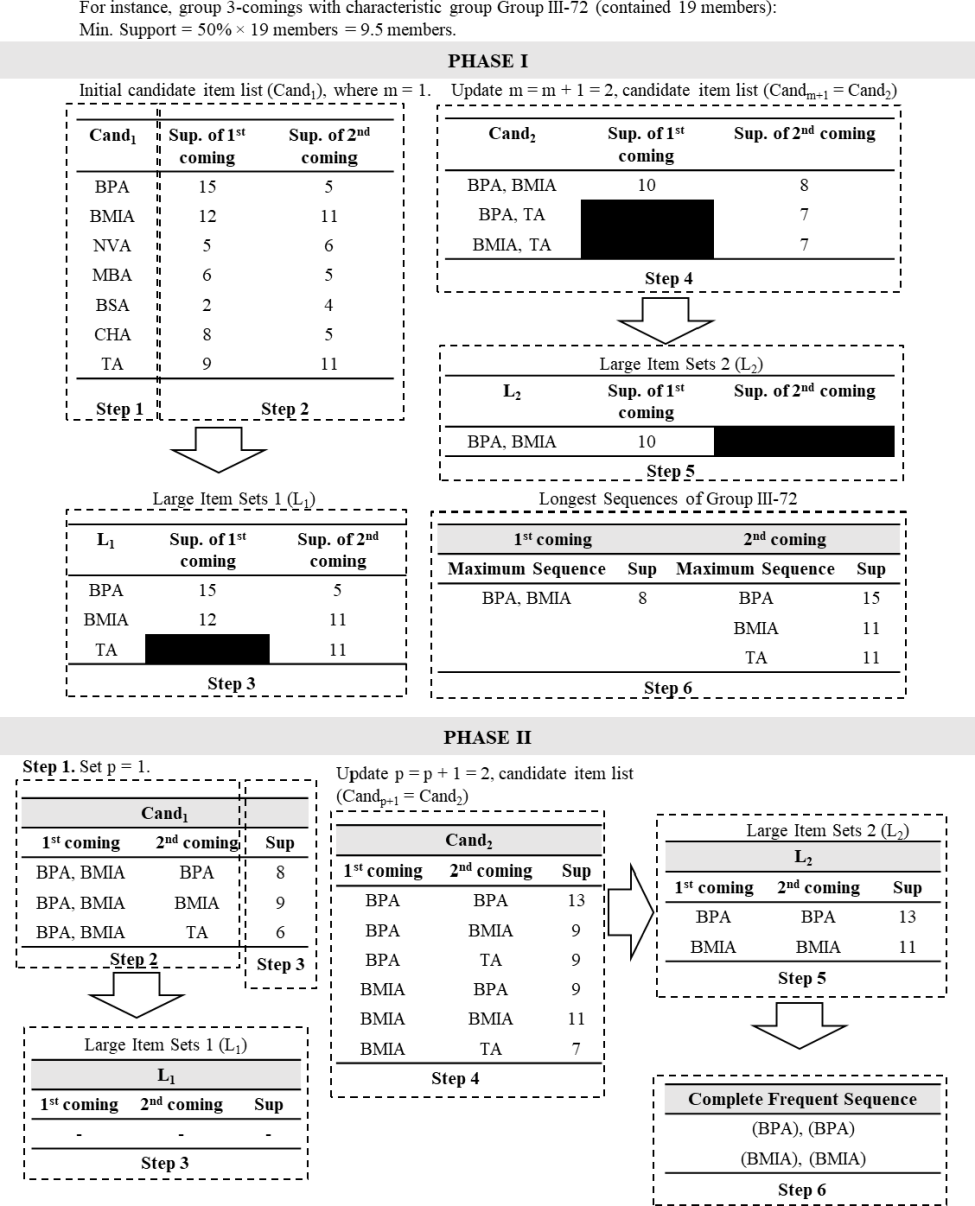
Illustration of two-phase sequential pattern mining (SPM).

After the final grouping result was obtained, the next step was extracting frequency patterns by employing SPM (Mooney & Roddick, 2013). This research tried to develop a new type of SPM method called a two-phase SPM. In the first phase of the method, frequency patterns were separately extracted by obtaining candidate sequence item sets of each characteristic group for each of the *c-* visits. The traditional priori-based SPM (Srikant & Agrawal, 1996) was employed in this phase by converting the dataset into candidate sequence item sets, in which procedures for each characteristic group are described in pseudocode in Table S5 and illustrated in Fig. 2.

### Mining association patterns

Regarding the complete frequent sequence patterns of each of the *c-* visit groups, the association mining method can be employed to obtain combinations of frequent sequence patterns which have high association values. The association mining method can be conducted using the following procedures (Ou-Yang, Agustianty & Wang, 2013).

#### Step 1. Defining health factor variable behaviors

The presence of health factor variables in each visit sequence for all *c-* visit groups is defined using specific notation. For instance, in the three-visit group, the notation *f*_*xy*_ represents the presence value of a health factor variable in the frequent sequence pattern, where *f* is the health factor variable, *x* is the presence of an abnormal value of variable *f* at the 1st visit (*x* = ‘1’ implies that the abnormal variable value is present, *x* = ‘0’ means otherwise), and *y* is the presence of an abnormal value of variable *f* at the 2nd visit (*x* = ‘1’ implies that the abnormal variable value is present, *x* = ‘0’ means otherwise). Note that the subscript letters follow the value of the frequency of visit sequences from the *c-* visit group, in which (*c* –1)*.* For instance, in the four-visit group, there are *c* –1 = 4 –1 = 3 visit sequences, which are further denoted by *f*_*xyz*_ and so forth.

***Example:***

Suppose the frequent sequence pattern item sets in the three-visit group consists of BPA (blood pressure abnormal) and NVA (neck vascular ultrasound abnormal). If the former variable behavior is showing BPA_10_*,* it implies that abnormal blood pressure was detected in the first visit, while in the second visit, blood pressure was normal. Henceforth, the variable behavior, BPA_10_, is denoted by the general behavior notation of *f*_10_. In addition, if the latter variable behavior is denoted by NVA_11_, then for the first and second visits, NVA continued. The general behavior notation for NVA_11_ is *f*_11_.

#### Step 2. Generating variable pair candidates

In this step, pairs of variable behaviors in a complete frequent sequence pattern are generated to represent the association between two variables in pairs. There is only one possible pair in the two-visit group, which is {*f*_1_}∧{*f*_1_}. For the three-visit group, six possible pairs of general behaviors can be generated: {*f*_11_}∧{*f*_11_}, {*f*_11_}∧{*f*_10_}, {*f*_11_}∧{*f*_01_}, {*f*_10_}∧{*f*_10_}, {*f*_10_}∧{*f*_01_}, and{*f*_01_}∧{*f*_01_}. Hence, variable pair candidates can be generated from the corresponding complete frequent sequence pattern of *c-* visits, in accordance with all possible pairs of general behaviors.

#### Step 3. Calculating the degree of association between variables

After all variable pair candidates were generated, the degree of association for each pair was measured. In this study, two symmetric measures (*Cosine* and *Jaccard*) were selected because the relation of each variable in a pair is symmetric. Therefore, there is no if-then relation in each pair. Azevedo and Jorge (2007) noted that *Cosine* and *Jaccard* are used to measure the degree of overlap between cases covered by each of the variables. *Jaccard* assesses the distance between two variables as the fraction of cases covered by both variables with respect to the fraction of cases covered by one of them Eq. (2). High values of *Jaccard* imply that those two variables are liable to cover the same cases. On the other hand, *Cosine* is used to measure the distance between variables when these are viewed as two binary vectors Eq. (3). Those two variables are considered to coincide if the value of *Cosine* is equal to 1; otherwise, those vectors have no overlap and the *Cosine* value is equal to 0.

(2) \begin{eqnarray*}& & Jaccard(A\cap B)= \frac{P(A\cap B)}{P(A)+P(B)+P(A\cap B)} \end{eqnarray*}

(3) \begin{eqnarray*}& & Cosine(A\cap B)= \frac{P(A\cap B)}{\sqrt{P(A)\times P(B)}} \end{eqnarray*}

where P(A) and P(B) respectively denote the probabilities that variables A and B appear in the dataset. In addition, P(A, B) represents the probability that variables A and B appear together in the dataset.

#### Step 4. Discovering variable associations

All pairs of variables were ranked according to *Cosine* and *Jaccard* values obtained from the previous step. A higher value implied that a pair of variables had a strong association. Therefore, those pairs might be considered as the reason why outpatients tended to return for a medical check-up.

## Results

According to results of the discriminant analysis, the dataset used in this study only consisted of two groups of visits (two-visit and three-visit groups). The parameter of the minimum support value in this study was 50%.

### Results of final data grouping of ABD variables

According to Table S3, there were some groups in which cardinality did not satisfy the cardinality threshold. Hence, merging such a group with another group, which was closest in distance, was necessary. Using SPSS software (SPSS, Chicago, IL, USA), the centroid coordinate of candidate merging groups was obtained. The distance between candidate merging groups was computed by means of the Euclidean distance. The merging process was iteratively performed until all groups’ cardinality satisfied the threshold. The group merging processes of the two-visit and three-visit groups from dataset are respectively illustrated in Figs. S1 and S2.

**Table 3:** Final grouping characteristics result of age, blood pressure (BP) history, and diabetes history variables.

Dataset	Group	Age	BP. history	Diabetes history	No. of members	Explained character	% Explained
Two-visits	II-23	Age 3	No	No	131	131	100%
	II-37	Age 1	No	No	204	195	96%
	II-39	Age 2	No	No	161	152	94%
	II-40	Age 4	No	No	95	88	93%
	II-41	Age 4	Yes	No	89	78	88%
	II-43	Age 1, 2, 3	No	Yes	24	24	100%
	II-44	Age 4	–	Yes	29	29	100%
	II-45	Age 1, 2, 3	Yes	No	139	139	100%
	II-46	Age 2, 3	Yes	Yes	13	13	100%
Three-visits	III-23	Age 3	No	Yes	11	11	100%
	III-29	Age 4	Yes	No	11	11	100%
	III-53	Age 2	No	No	27	24	89%
	III-60	Age 1	No	No	28	24	86%
	III-66	Age 1 → 2 Age 2 →3	No	No	19	15	79%
	III-68	Age 4 Age 3 →4	No	No	23	22	96%
	III-71	Age 3 Age 2 →3	Yes	No	26	13	50%
	III-72	Age 2 Age 1	Yes	No	19	18	95%

The final characteristic of the ABD-based grouping result and the percentage value of the explained characteristic are presented in Table 3. Group characteristics, which are presented in columns three to five of Table 3, are defined by identifying characteristics of a group’s members that mostly appear in the corresponding merged group. The explained character denotes the total number of members which contained the corresponding group characteristic.

Furthermore, an evaluation of the significant difference between groups is required. The purpose is to ensure that ABD-based grouping results actually split the data in accordance with the similarity. Box’s M test in the discriminant analysis was employed to estimate the within-group covariance, with a critical value of 50% (Hair et al., 2006), and was performed using SPSS software. Table 4 presents results of the discriminant analysis, in which significant values of group characteristics from the two-visit and three-visit data records were <0.05. This implies that the grouping result was good. However, an error result occurred for the four-visit data records because they contained a large number of variables with a small number of data. In the discriminant analysis, the more variables used, the more data are required. Henceforth, the two-visit and three-visit group datasets were used to generate frequent sequence patterns and association mining.

### SPM analysis and results

SPM was conducted on a cerebrovascular health medical records dataset to generate hidden knowledge, which could possibly represent outpatients’ re-visit behavior patterns toward regular medical check-ups. In general, the generated results of frequent sequence patterns can be analyzed by identifying characteristics of an outpatient’s return based on different personal ABD health histories.

For instance, in the frequency sequence pattern results of two-visit data records presented in Table 5, groups II-23, II-39, and II-40 were considered to be middle-aged people (aged over 47 years), with no history of abnormal blood pressure or diabetes. People in group II-40 (aged over 59 years) were more likely to come in for a second visit because of an abnormal blood pressure or abnormal cholesterol which occurred at their first clinical visit. The former frequent sequence pattern contributed the highest support value of 63%, while the support value for the latter was 61%. Another high support frequent pattern showed that 62% of middle-aged (aged 53–58 years) people in group II-23 came for a second visit after having been diagnosed with abnormal cholesterol.

**Table 4:** Statistical results of the discriminant analysis.

Group of *c*-visits	No. of data records	No. of groups	No. of variables	Sig.
2-visits	885	9	8	0.000
3-visits	165	8	16	0.000
4-visits	49	4	24	Error

**Table 5:** Complete frequent sequence patterns for all characteristic groups in the two-visit dataset.

Group	Age	BP_history	Dia_history	Member	Frequent Seq.	Sup. count	Sup.
II-23	Age 3	No	No	131	BPA	75	57%
					CHA	81	62%
II-37	Age 1	No	No	204	–	–	–
II-39	Age 2	No	No	161	BPA	86	53%
					CHA	87	54%
II-40	Age 4	No	No	95	BPA	60	63%
					NVA	54	57%
					CHA	58	61%
II-41	Age 4	Yes	No	89	BPA NVA	49	55%
					BMIA	52	58%
					MBA	45	51%
					CHA	50	56%
II-43	Age 1, 2, 3	No	Yes	24	BSA TA	13	54%
					BMIA	14	58%
II-44	Age 4	–	Yes	29	BPA NVA BSA	17	59%
					BPA MBA	16	55%
					BMIA	15	52%
II-45	Age 1, 2, 3	Yes	No	139	BPA BMIA	79	57%
					CHA	80	58%
					TA	77	55%
II-46	Age 2,3	Yes	Yes	13	BPA BMIA BSA	8	62%
					BPA NVA	7	54%
					BMIA TA	7	54%
					NVA BSA	7	54%
					BSA TA	7	54%

**Notes.**

TITLE BP blood pressure BPA BP abnormal Dia diabetes ABD age, blood pressure history, and diabetes history CHA cholesterol abnormal NVA neck vascular ultrasound abnormal MBA magnetic resonance imaging of the brain abnormal BSA blood sugar abnormal TA triglycerides abnormal

On the other hand, results also showed that outpatients with the characteristics of group II-44, i.e., being aged over 59 years and with a history of diabetes, were more likely to re-visit the clinic for a second time. The frequency sequence pattern contained abnormal blood pressure, abnormal blood sugar, as well as an abnormal neck vasculature ultrasound on the first visit, and had a 59% support value. Results of group II-44 imply that the pattern occurred often, and the corresponding outpatients could probably be diagnosed with a stroke in accordance with an abnormal result from the radiology test, especially through the neck vasculature ultrasound. Based on this condition, people of this age and in a poor health condition are at high risk of stroke according to the domain knowledge. Moreover, physicians could probably prepare appropriate medications for outpatients or suggest special treatments such as an MRI brain test to ensure the diagnosis, if necessary.

In Table S6, groups III-53, III-60, and III-68 showed no frequent sequence pattern. This implies that with no history of hypertension or diabetes, and no abnormal conditions in their health factor variables even on their second visit, it is basically their habit to seek regular medical check-ups. This indicates that those people can maintain their health from a young age and into old age.

Regarding the SPM results of the three-visit group presented in Table S6, we highlighted some patterns which had high support values. Groups III-66 and III-72 showed interesting frequent sequence patterns which were quite similar to one another. The former group contained changes in characteristic in terms of age, from Age1 to Age2 and Age2 to Age3, with no history of hypertension or diabetes. In group III-72, people under age 26–52 years with a history of hypertension were more likely to re-visit the clinic for a medical check-up and to find the same result on each visit. For instance, if they found abnormal blood pressure or abnormal BMI on the first and second visits, they would be more likely to visit the clinic a third time. The support value of these patterns was up to 68%. Learning by this condition, the physician could probably begin suggesting that outpatients go for a consultation with a nutritionist on the third visit, as a marketing purpose of medical services to take care of the abnormal BMI condition. In addition, they could also suggest other medications to outpatients, whose blood pressure remained in an abnormal condition through their second visit.

### Results and analysis of association mining

Results of association mining that consisted of a single variable can be seen in columns 2 and 4 of Table 6 and Table S7 for the two-visit and three-visit groups, respectively. In general, abnormal blood pressure, abnormal BMI, and an abnormal neck vasculature ultrasound test were single variables that were found to be very indicative of the re-visit behavior in the two-visit and three-visit datasets, due to their large support counts. However, considering a single variable directly as outpatients’ re-visit behavior characteristics might be less useful, because the knowledge is too general. Hence, in this section, an analysis of the results focuses on some pairs of health factor variables that can be measured to determine pairs of variables which have a high association leading to re-visit behavior patterns. Each pair of health factor variables can be identified in accordance with the general behaviors of each of the *c*-visits and its degree of association (*Jaccard* and *Cosine* measurements). As presented in Table 6 and Table S7, every pair represents an association between variables that occurred in frequent item sets of the *c*-visit group.

**Table 6:** Degree of association under general behavior for the two-visit dataset.

Var. Pair	*f*_1_	Sup.	*f*_1_	Sup.	*f*_1_∧*f*_1_	Sup.	Cosine	Jaccard
II-1	BPA	176	BMIA	94	BPA_1_∧ BMIA_1_	87	0.676	0.475
II-2	BPA	176	NVA	80	BPA_1_∧ NVA_1_	73	0.615	0.399
II-8	BSA	52	TA	27	BSA_1_∧ TA_1_	20	0.534	0.339
II-7	NVA	80	BSA	52	NVA_1_∧ BSA_1_	24	0.372	0.222
II-4	BPA	176	BSA	52	BPA_1_∧ BSA_1_	25	0.261	0.123
II-3	BPA	176	MBA	16	BPA_1_∧ MBA_1_	16	0.302	0.091
II-6	BMIA	94	TA	27	BMIA_1_∧ TA_1_	7	0.139	0.061
II-5	BMIA	94	BSA	52	BMIA_1_∧ BSA_1_	8	0.114	0.058

**Notes.**

TITLE Sup. support BPA blood pressure abnormal BSA blood sugar abnormal NVA neck vascular ultrasound abnormal BMIA body-mass index abnormal

In general, behavior {*f*_1_}∧{*f*_1_} of the two-visit group, a variable abnormal result of blood pressure paired with an abnormal BMI, at the first visit, showed a quite high association degree with the outpatients’ re-visit behavior. The variable pair was considered to coincide with the *Cosine* value of 67.6% and covered the re-visit behavior pattern with 47.5% for the *Jaccard* value. On the other hand, variable blood pressure also had quite a high association when it was paired with the radiology diagnosis. The value of the cosine for BPA_1_∧NVA_1_ was 61.5%, which was two-times higher than BPA_1_∧MBA_1_. This means that patients with the pairing of abnormal blood pressure and an abnormal neck vasculature ultrasound result were more likely to represent the behavior than MRI of the brain. The reason might be that ultrasound is available in many places throughout the country and is less expensive than MRI; hence it is still reasonable to perform a radiology diagnosis on the first visit. Another high association in the two-visit dataset was found in the variable pair of abnormal blood sugar (indicating diabetes) and abnormal triglycerides (indicating hypertension) on the first visit. The degree of association for this pair reached a *Cosine* value of 53.4%, which implies that those two variables coincided but covered the re-visit behavior pattern by only 33.9% according to the *Jaccard* value. However, a high degree of association was obtained in the case of the three-visit dataset, in which abnormal blood sugar occurred along with abnormal BMI, both of which are related to diabetes.

A similar result was also shown in the variable pair III-1-2, from general behavior {f11}∧{f11} of the three-visit group presented in Table S7, with respective *Cosine* and *Jaccard* values of 43.3% and 18.8%. Nevertheless, in the three-visit group, outpatients with abnormal blood pressure who had an abnormal MRI of the brain result on their second visit had a higher degree of association than those outpatients who continuously had abnormal neck vascular ultrasound results in every visit. In this case, the physician could recommend that outpatients have an MRI to follow-up on the abnormal findings and get a more-detailed view. Once the results show an abnormality, outpatients tend to begin scheduling regular medical check-ups to monitor the progress of their health conditions.

Our findings show that variable pairs with the highest degree of association were found in general behavior {f11}∧{f01} followed by {f10}∧{f01}. Those variable pairs had *Cosine* and *Jaccard* values of 100%. The former general behavior implies that the presence of an abnormal variable *f* still continued in the subsequent visit, while the latter shows the change in variable *f* from normal at the first visit to abnormal in the second visit. Those were associated with outpatients’ re-visit behavior patterns. In particular, a patient who had an abnormal blood pressure tended to begin monitoring their health condition only if it was paired with obesity and/or abnormal blood sugar. The measurement values show that each variable pair in general behavior {f11}∧{f01} coincided with and covered 100% of the re-visit behavior pattern.

Moreover, variable pair III-3-1 emphasizes that any patient who receives an MRI of the brain will tend to return for another medical check-up. This interpretation was also supported by variable pair III-2-2 to variable pair III-2-5 in general behavior {f11}∧{f01}, which contained either MBA or NVA variables. This implies that most outpatients will begin monitoring their health after receiving a radiology diagnosis such as an MRI of the brain or a neck vasculature ultrasound test. Even though the degrees of association of these variable pairs were not as high as general behavior {f11}∧{f01}, which was only around 50%∼70% of the *Cosine* and *Jaccard* measures, it still can be implied that a radiology diagnosis plays an important role in the association with outpatients’ re-visit behavior patterns. Whether the MBA or NVA continuously occurs in every visit in the sequence or only in the first or second visit, these features can encourage outpatients to return to check the progress of their health conditions or to ensure that the result is actually a matter of concern.

### Result validation

Evaluation of the obtained results in clinical practice is often based on interpretations by domain experts. In this work, we created a questionnaire with 10 questions to determine the degree of agreement among experts toward our findings (Appendix S1). Those experts were from six different divisions such as general medicine, general surgery, neurology, plastic surgery, rehabilitation, and pediatrics. An index of inter-rater agreement in statistical methods, the so-called content validity index (CVI) (Horgas et al., 2008; Jakobsson & Westergren, 2005; Larsson et al., 2015) and the modified Kappa value (Larsson et al., 2015) were used to measure agreement for ordinal data. The experts were asked to what extent they agreed with each item of our findings on a 5-point Likert scale, which ranged from 1 (strongly disagree) to 5 (strongly agree). For each item, the item CVI (I-CVI) was computed as the number of experts giving a rating of either 4 (agree) or 5 (strongly agree), divided by the total number of experts. The evaluation criteria of the agreement, proposed by Landis & Koch, 1977, were used to interpret the strength of agreement beyond chance based on a modified Kappa value (k*). A value of k* <0 is poor (P); k* = 0∼0.20 is slight (Sl); k* = 0.21∼0.40 is considered fair (F); k* = 0.41∼0.60 is moderate (M); k* = 0.61∼0.80 is substantial (S); and k* = 0.81∼1.00 is almost perfect (A).

Table 7 presents the overall degree of agreement of 10 items by 16 experts. According to the results, the experts tended to agree with items that focused on re-visit behavior patterns of middle-age and old people, who underwent a regular medical check-up, due to their experience with hypertension and/or diabetes as well as abnormalities found on the radiology test. Those items obtained the strength of agreement of “substantial” to “almost perfect” in the range of 60%–100%. This situation seems to be common knowledge among physicians. On the other hand, evaluation criteria for items Q1, Q3, and Q10 were slight (Sl), in which the experts found this behavior to be uncommon in their knowledge. However, according to our dataset, the aforementioned items were a fact of outpatients’ re-visit behavior patterns that might still be uncommon knowledge for experts, yet could be expected to provide useful insights for medical services to provide appropriate treatment and increase the service quality. Additionally, some of the items from Q6 to Q9 represented the association of variable pairs, while some others represented the radiology test and its association with blood pressure or blood sugar. The strength of agreement among experts toward these items of our findings was considered to be fair (F) or moderate (M) in the range of 20%–60%.

**Table 7:** Degree of agreement on 10 items by 16 experts: items rated 4 or 5 on a 5-point agreement scale.

Item	Q1	Q2	Q3	Q4	Q5	Q6	Q7	Q8	Q9	Q10
Expert-1	–	√	–	√	–	–	√	√	–	–
Expert-2	–	√	–	–	–	–	–	–	√	–
Expert-3	–	√	–	√	√	–	–	√	√	–
Expert-4	–	√	–	–	√	–	–	–	√	–
Expert-5	–	√	–	√	√	√	√	√	–	–
Expert-6	–	√	–	√	√	√	√	√	–	–
Expert-7	–	√	–	–	–	–	–	–	√	√
Expert-8	–	√	–	√	√	√	√	√	–	–
Expert-9	√	–	–	√	√	√	–	–	√	–
Expert-10	–	√	√	–	√	√	√	–	√	–
Expert-11	–	√	–	√	√	–	√	√	–	–
Expert-12	–	–	–	√	√	–	–	√	–	–
Expert-13	√	–	–	–	√	–	–	√	√	–
Expert-14	–	√	–	√	√	–	√	–	–	–
Expert-15	–	√	√	√	√	–	–	√	–	–
Expert-16	–	√	–	√	√	√	–	–	√	–
Expert in agreement	2	14	2	12	14	7	8	10	9	1
Item CVI (I-CVI)	0.13	0.88	0.13	0.75	0.88	0.44	0.50	0.63	0.56	0.06
Average I-CVI	0.49									
Kappa value (*k*^∗^)	0.12	0.87	0.12	0.74	0.87	0.32	0.38	0.57	0.47	0.06
Strength of agreement	*Sl*	*A*	*Sl*	*S*	*A*	*F*	*F*	*M*	*F*	*Sl*

**Notes.**

TITLE CVI content validity index Sl slight A almost perfect S substantial F fair M moderate

## Discussion

According to Table 5, most of the frequent patterns showed that outpatients’ were most likely to come for a second visit if they found an abnormality with any health factor variable, especially blood pressure and cholesterol. Therefore, at the second visit, the physician could suggest a preventive measure, such as regular medication for hypertension, diabetes, and/or cholesterol, because they were unlikely to suffer from a stroke at that point. Additionally, physicians should be concerned whenever an outpatient exhibits a pattern in which several abnormal health factors occur together at the first visit. It might be considered likely to cause a possible serious disease. Hence, medical practitioners can prepare appropriate treatments for outpatients with the corresponding pattern, such as a suggestion for a consultation with a nutritionist to take care of an abnormal BMI or proper medication for diabetes and hypertension, at their second visit.

Additional insights that can be derived from Table S6 is that middle-aged people have a high probability of worsening blood pressure or BMI, but they lack concern for their health. Most people in this age range will begin to be concerned about their health only if any abnormalities in the variables related to hypertension or diabetes are found, despite having no previous history of those. In addition, abnormal results from a radiology diagnosis (such as neck vascular ultrasound or even MRI of the brain) in the elderly contribute to their re-visit behavior patterns. This probably occurred because they are more likely to begin to be concerned about their brain condition and recognize the possibility of an increased risk of stroke. Thus, the elderly will begin to seek regular medical check-ups. At this point, medical service providers can begin suggesting preventive measures or treatments for outpatients with stroke.

In general, outpatients were aware of their blood pressure. Most of the pairs of variables contained abnormal blood pressure as one of the items, whether this abnormal condition occurred on the first visit or later. For instance, in general behavior {f10}∧{f01}, the pairing of abnormal blood pressure and abnormal MRI of the brain had a very high degree of association. This implies that if abnormal blood pressure becomes normal, whereas a new abnormal MRI result of the brain is found on the second visit, or vice versa, outpatients will tend to return for a third visit to confirm whether or not the abnormal result from the first or second visit was valid. In terms of marketing purposes, medical service providers can consider the behavior patterns examined in this paper to increase outpatients’ awareness of several possible medical conditions that might arise on subsequent visits and encourage them to take preventive measures or suggest other medical treatments offered by the physicians. There was no association between variables that had general behavior {f10}∧{f01}, which means that this behavior seldom appeared in outpatient re-visit patterns of our dataset.

## Conclusions

To the best of our knowledge, few studies have investigated characteristics of outpatients’ re-visit behavior patterns from a cerebrovascular health examination dataset. In this research, sequential pattern mining was employed to mine patterns that might identify outpatients’ re-visit behavior patterns. The data were divided into several groups in accordance with personal variables (age, hypertension history, and diabetes history) so that the generated patterns would give more-specific information on those characteristics. For generalization, we derived some pairs of variables that had high degrees of association using an association mining approach.

Results showed that in general, most of the re-visit behavior patterns were affected by abnormal blood pressure and/or a radiology diagnosis (i.e., an MRI or neck vascular ultrasound), because that abnormal variable often appeared in a complete frequent sequence. Furthermore, associated pattern mining showed consistent results, in which some variable pairs that had a high degree of association were identified as characteristics most likely to influence outpatients to return. Our findings also support the fact that people who have great concern about their health and regularly seek medical check-ups as a habit will maintain a healthy condition as they age. The more details medical practitioners know about outpatients’ re-visit behavior patterns, the better they can provide appropriate treatments for expected outpatients’ visits in the future. This can be one of several possible efforts to increase the quality of medical/health services for marketing purposes.

##  Supplemental Information

10.7717/peerj.5183/supp-1 Supplemental Information 1 Supplemental Figures

10.7717/peerj.5183/supp-2 Supplemental Information 2 Supplemental Tables

10.7717/peerj.5183/supp-3 Appendix S1 List of questions for experts

10.7717/peerj.5183/supp-4 Supplemental Information 3 Raw data

## Additional Information and Declarations

### Data Availability

The following information was supplied regarding data availability: The raw data are provided in a Supplemental File.
