# Supplementary material for: Applying sequential pattern mining to investigate cerebrovascular health outpatients’ re-visit patterns
Source: PeerJ. 2018 Jul 9;6:e5183. doi: 10.7717/peerj.5183 (PMC6042480; doi:10.7717/peerj.5183)
Supplement: Supplemental Information 1 [file peerj-06-5183-s001.pdf]

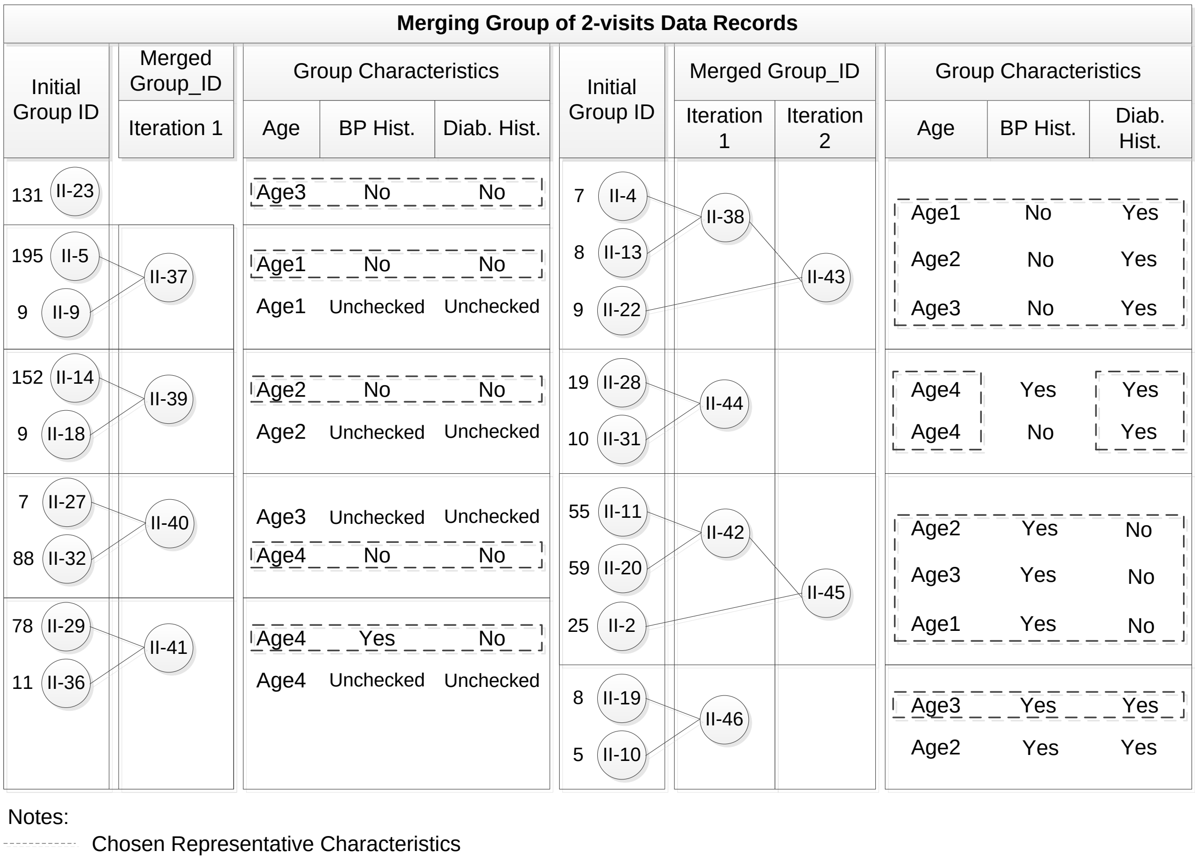

Figure S1. Group Merging Process for 2-visits Data Records

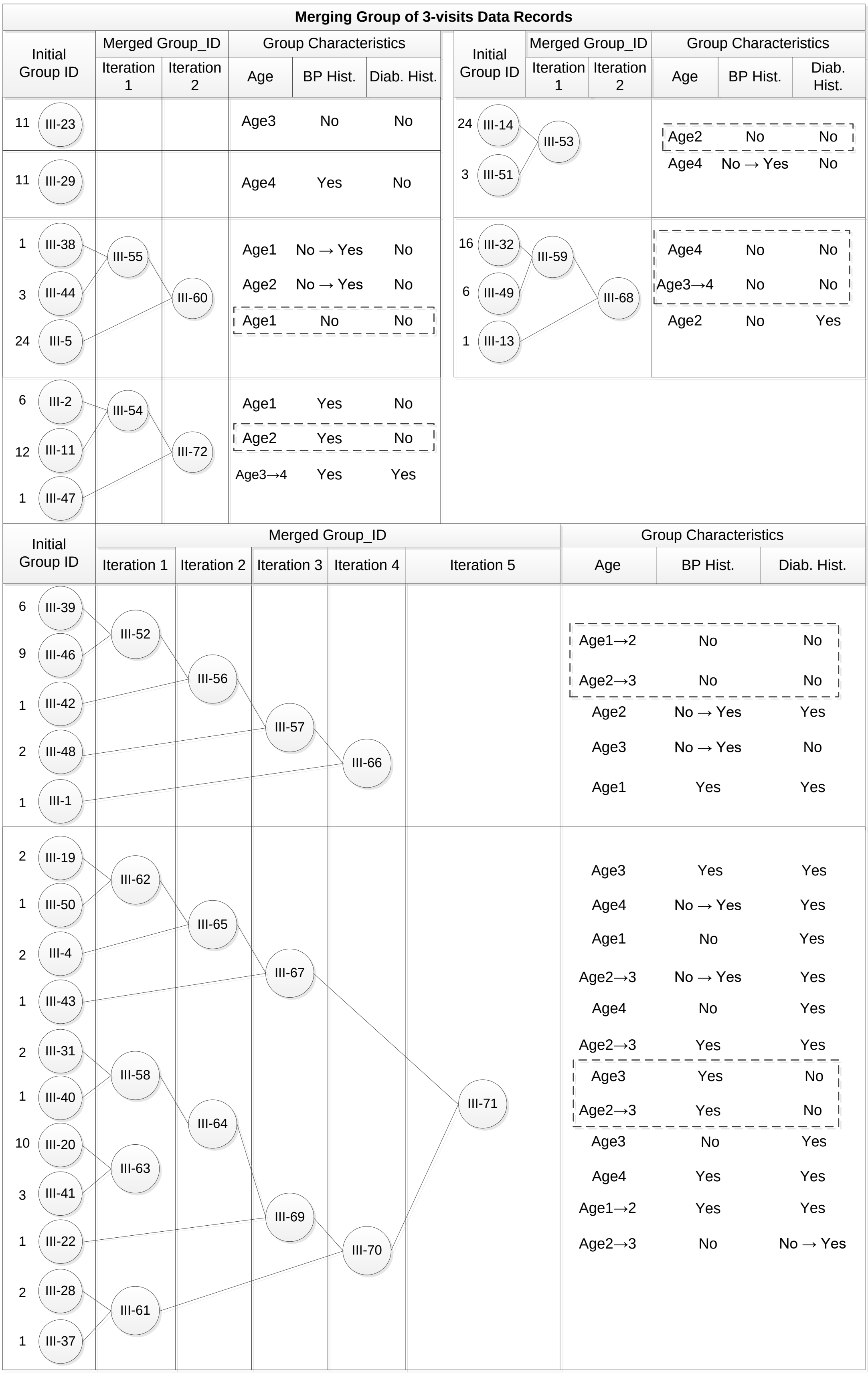

Notes:

Chosen Representative Characteristics

Figure S2. Group Merging Process for 3-visits Data Records
