# Supplementary material for: Applying sequential pattern mining to investigate cerebrovascular health outpatients’ re-visit patterns
Source: PeerJ. 2018 Jul 9;6:e5183. doi: 10.7717/peerj.5183 (PMC6042480; doi:10.7717/peerj.5183)
Supplement: Supplemental Information 2 [file peerj-06-5183-s002.pdf]

Table S1. Initial Combinations of ABD-Group Characteristics (before changing group characteristics)

**Group**	**ABD Variables**			**Group**	**ABD Variables**		
	**Age**	**BP_history**	**Diabetes_history**		**Age**	**BP_history**	**Diabetes_history**
**g-1**	Age1	Yes	Yes	**g-19**	Age3	Yes	Yes
**g-2**			No	**g-20**			No
**g-3**			Uncheck	**g-21**			Uncheck
**g-4**		No	Yes	**g-22**		No	Yes
**g-5**			No	**g-23**			No
**g-6**			Uncheck	**g-24**			Uncheck
**g-7**		Uncheck	Yes	**g-25**		Uncheck	Yes
**g-8**			No	**g-26**			No
**g-9**			Uncheck	**g-27**			Uncheck
**g-10**	Age2	Yes	Yes	**g-28**	Age4	Yes	Yes
**g-11**			No	**g-29**			No
**g-12**			Uncheck	**g-30**			Uncheck
**g-13**		No	Yes	**g-31**		No	Yes
**g-14**			No	**g-32**			No
**g-15**			Uncheck	**g-33**			Uncheck
**g-16**		Uncheck	Yes	**g-34**		Uncheck	Yes
**g-17**			No	**g-35**			No
**g-18**			Uncheck	**g-36**			Uncheck

Table S2. Additional Groups of Characteristic for 3-visits and 4-visits Dataset

**Group Characteristic**						
**Group**	**3-visits Dataset**		**Group**	**4-visits Dataset**		
	**1st Visit**	**2nd Visit**		**1st Visit**	**2nd Visit**	**3rd Visit**
**III-37**	g-1	g-10	**IV-37**	g-5	g-14	g-14
**III-38**	g-5	g-2	**IV-38**	g-9	g-2	g-11
**III-39**	g-6	g-14	**IV-39**	g-9	g-18	g-14
**III-40**	g-10	g-19	**IV-40**	g-10	g-10	g-19
**III-41**	g-11	g-20	**IV-41**	g-11	g-20	g-20
**III-42**	g-13	g-10	**IV-42**	g-13	g-22	g-22
**III-43**	g-13	g-19	**IV-43**	g-14	g-13	g-13
**III-44**	g-14	g-11	**IV-44**	g-14	g-14	g-23
**III-45**	g-14	g-22	**IV-45**	g-14	g-23	g-23
**III-46**	g-14	g-23	**IV-46**	g-18	g-14	g-14
**III-47**	g-19	g-28	**IV-47**	g-22	g-31	g-31
**III-48**	g-23	g-20	**IV-48**	g-23	g-23	g-20
**III-49**	g-23	g-32	**IV-49**	g-23	g-23	g-32
**III-50**	g-31	g-28	**IV-50**	g-23	g-32	g-32
**III-51**	g-32	g-29	**IV-51**	g-27	g-27	g-23
			**IV-52**	g-27	g-27	g-32
			**IV-53**	g-32	g-29	g-29
			**IV-54**	g-32	g-31	g-31
			**IV-55**	g-36	g-29	g-29
			**IV-56**	g-36	g-32	g-31

Table S3. Initial ABD-Group Characteristics Result

**2-visits Dataset**		**3-visits Dataset**				**4-visits Dataset**			
Group	Member	Group	Member	Group	Member	Group	Member	Group	Member
**II-2**	25	**III-1**	1	**III-42**	1	**IV-2**	1	**IV-47**	1
**II-4**	7	**III-2**	6	**III-43**	1	**IV-5**	1	**IV-48**	1
**II-5**	195	**III-5**	24	**III-44**	3	**IV-10**	1	**IV-49**	1
**II-9**	9	**III-11**	12	**III-45**	1	**IV-11**	1	**IV-50**	4
**II-10**	5	**III-13**	1	**III-46**	9	**IV-14**	4	**IV-51**	1
**II-11**	55	**III-14**	24	**III-47**	1	**IV-23**	3	**IV-52**	2
**II-13**	8	**III-19**	2	**III-48**	2	**IV-27**	1	**IV-53**	2
**II-14**	152	**III-20**	10	**III-49**	6	**IV-28**	1	**IV-54**	1
**II-18**	9	**III-22**	1	**III-50**	1	**IV-32**	3	**IV-55**	1
**II-19**	8	**III-23**	11	**III-51**	3	**IV-37**	3	**IV-56**	1
**II-20**	59	**III-28**	2			**IV-38**	1		
**II-22**	9	**III-29**	11			**IV-39**	1		
**II-23**	131	**III-31**	2			**IV-40**	1		
**II-27**	7	**III-32**	16			**IV-41**	1		
**II-28**	19	**III-37**	1			**IV-42**	1		
**II-29**	78	**III-38**	1			**IV-43**	1		
**II-31**	10	**III-39**	6			**IV-44**	2		
**II-32**	88	**III-40**	1			**IV-45**	4		
**II-36**	11	**III-41**	3			**IV-46**	3		
**Member <10**	8	**Member <10**		12		**Member <10**		29	

Table S4. Experimental Protocol of Group Merging and Discriminant Analysis

Input: Set of medical data records $\boldsymbol{M} = \{\boldsymbol{m_1}, \boldsymbol{m_2}, \ldots, \boldsymbol{m_R}\}$ and set of group characteristics $\boldsymbol{G} = \{\boldsymbol{g_1}, \boldsymbol{g_2}, \ldots, \boldsymbol{g_K}\}$ obtained from Table 5 (Section 3.2.2).
Output: Final Grouping Characteristic of ABD Variables

1: Specify each medical data record *m* in accordance with *G*
2: Calculate the distance between the centroids of the groups by means of Euclidean distance formula (Equation 2):

$$dist(p,q) = \sqrt{\sum_{d=1}^{D} (p_d - q_d)^2} \qquad (1)$$

3: Identify the cardinality of each group in *G*
for each $k \in K$
  if $|\boldsymbol{g_k}| < \boldsymbol{card_{min}}$ **then**
    Merge corresponding g_k with another groups which are closest in distance
  end
end
4: Update $\boldsymbol{G}$
5: Iterate between 1 to 4 until all the group cardinality satisfy the $\boldsymbol{card_{min}}$
6: Return final *G*
7: Check for significant differences between groups according to *Box's M* test result in discriminant analysis (Hair et al. 2006)
if significant value of *Box's M* test < 0.05 **then**
  Grouping result is good
else
  Find alternative statistical methods
end
8: Return final *G*

Notations:

$\boldsymbol{M}$	: Set of medical data records *m*, indexed by $r = 1, 2, \ldots, R$
$\boldsymbol{R}$	: Total number of records in the dataset
$\boldsymbol{m_r}$	: Set of data records *r* contained of *D*-dimensions
$\boldsymbol{D}$	: Total number of dimensions in the dataset, indexed by $d = 1, 2, \ldots, D$
$\boldsymbol{G}$	: Set of group characteristics g (Table 5), indexed by $k = 1, 2, \ldots, K$
$\lvert\boldsymbol{g_k}\rvert$	: Total number of members (cardinality) of group $\boldsymbol{g_k}$
$\boldsymbol{card_{min}}$	: Cardinality threshold
$\boldsymbol{p_d}$	: Coordinate centroid of the 1st group in dimension *d*
$\boldsymbol{q_d}$	: Coordinate centroid of the 2nd group in dimension *d*

Table S5. Pseudocode for 2-Phase Sequential Pattern Mining (SPM)

Input: Set of medical data records $\boldsymbol{M} = \{\boldsymbol{m_1}, \boldsymbol{m_2}, \dots, \boldsymbol{m_R}\}$ and minimum support $(\boldsymbol{\alpha_{min}})$
Output: Complete Frequent Sequence Patterns

1: Sort *M* based on patient ID in ascending order
2: **begin** Phase 1, Set $m = 1$
3: Set all health factor variables in category 'abnormal' as $Cand_m = Cand_1$ (initial 1-item candidate sequence)
4: Calculate support value (α) for each candidate item in C_m in every visit history
  if $\alpha \geq \alpha_{min}$
    include the corresponding item into L_m
  end
5: Update $\{L_m\}$
6: Identify all possible combination of (*m*+1) items from candidate items in L_m as $Cand_{m+1}$
7: Set $m+1 = m$ and iterate between 4 to 7 until L_m is empty
8: Find L_m with maximum length of itemset
9: **return** L_m
10: **end** Phase 1
11: **begin** Phase 2, Set $p = 1$
12: Identify all possible combination items from LS_m as $Cand_p = Cand_1$ (initial 1-item candidate sequence)
13: Calculate support value (α) for each candidate item in $Cand_p$ in every visit history
  if $\alpha \geq \alpha_{min}$
    include the corresponding item into L_p
  end
14: Update $\{L_p\}$
15: Identify all possible combination of (*p*+1) items from candidate items in $Cand_p$
16: Set $p+1 = p$ and iterate between 13 to 16 until $Cand_p$ is empty
17: Find L_p with maximum length of itemset
18: **return** L_p
19: **end** Phase 2

Notations:

$\boldsymbol{M}$	: Set of medical data records m, indexed by $r = 1, 2, \dots, R$
$\boldsymbol{R}$	: Total number of records in the dataset
$\boldsymbol{\alpha_{min}}$	: Support threshold
$\boldsymbol{\alpha}$	: Support value
$\boldsymbol{L}$	: Large Itemset
$\boldsymbol{m, p}$	: Index for size of itemset
$\boldsymbol{Cand}$	: Possible candidate items

Table S6. Complete Frequent Sequence Patterns for All Characteristic Groups in 3-visits Dataset

Group	Age	BP_hist	Dia_hist	Member	Frequent Pattern			
					1st visit	2nd visit	Sup count	Sup
**III-23**	Age 3	No	No	11	CHA	CHA	6	55%
**III-29**	Age 4	Yes	No	11	BPA NVA MBA	NVA	6	55%
					BPA BMIA	BPA	6	55%
					BPA NVA	BPA NVA	6	55%
					BPA	BPA MBA	6	55%
					BPA NVA	MBA	6	55%
					BPA MBA	BPA	6	55%
					BPA MBA	MBA	6	55%
					NVA MBA	BPA	6	55%
**III-53**	Age 2	No	No	27	-	-		-
**III-60**	Age 1	No	No	28	-	-		-
**III-66**	Age1->2	No	No	19	BPA	BPA	10	53%
	Age2->3				BMIA	BMIA	11	58%
**III-68**	Age 4	No	No	23	-	-		-
	Age3->4							
**III-71**	Age 3	Yes	No	26	BMIA BSA	BPA BMIA BSA	14	54%
	Age2->3				BPA BMIA	BPA BMIA	13	50%
					BPA BSA	BPA BSA	13	50%
**III-72**	Age 2	Yes	No	19	BPA	BPA	13	68%
	Age 1				BMIA	BMIA	11	58%

Table S7. Degree of Association Under General Behavior for 3-visits Dataset

***General Behavior:* {f_{11}}^{f_{11}}**

Var. Pair	f_{11}	Sup	f_{11}	Sup	f_{11}^f_{11}	Sup	Cosine	Jaccard
**III-1-4**	BMIA	27	BSA	27	$BMIA_{11}$^BSA_{11}	14	0.519	0.350
**III-1-1**	BPA	32	BMIA	27	BPA_{11}^$BMIA_{11}$	13	0.442	0.283
**III-1-3**	BPA	32	BSA	27	BPA_{11}^BSA_{11}	13	0.442	0.283
**III-1-2**	BPA	32	NVA	6	BPA_{11}^NVA_{11}	6	0.433	0.188

***General Behavior:* {f_{11}}^{f_{10}}**

Var. Pair	f_{11}	Sup	f_{10}	Sup	f_{11}^f_{10}	Sup	Cosine	Jaccard
**III-2-1**	BPA	12	BMIA	6	BPA_{11}^$BMIA_{10}$	6	0.707	0.500
**III-2-4**	NVA	6	MBA	12	NVA_{11}^MBA_{10}	6	0.707	0.500
**III-2-5**	MBA	6	BPA	12	MBA_{11}^BPA_{10}	6	0.707	0.500
**III-2-2**	BPA	12	MBA	12	BPA_{11}^MBA_{10}	6	0.500	0.333
**III-2-3**	NVA	12	BPA	12	NVA_{11}^BPA_{10}	6	0.500	0.333

***General Behavior:* {f_{11}}^{f_{01}}**

Var. Pair	f_{11}	Sup	f_{01}	Sum	f_{11}^f_{01}	Sup	Cosine	Jaccard
**III-3-1**	BPA	6	MBA	6	BPA_{11}^MBA_{01}	6	1.000	1.000
**III-3-2**	BMIA	14	BPA	14	$BMIA_{11}$^BPA_{01}	14	1.000	1.000
**III-3-3**	BSA	14	BPA	14	BSA_{11}^BPA_{01}	14	1.000	1.000

***General Behavior:* {f_{10}}^{f_{10}}**

Var. Pair	f_{10}	Sup	f_{10}	Sum	f_{10}^f_{10}	Sup	Cosine	Jaccard
**III-4-1**	BPA	12	NVA	12	BPA_{10}^NVA_{10}	6	0.500	0.333
**III-4-2**	BPA	12	MBA	12	BPA_{10}^MBA_{10}	6	0.500	0.333
**III-4-3**	NVA	12	MBA	12	NVA_{10}^MBA_{10}	6	0.500	0.333

***General Behavior:* {f_{10}}^{f_{01}}**

Var. Pair	f_{10}	Sup	f_{01}	Sum	f_{10}^f_{01}	Sup	Cosine	Jaccard
**III-5-1**	BPA	6	MBA	6	BPA_{10}^MBA_{01}	6	1.000	1.000
**III-5-4**	MBA	6	BPA	6	MBA_{10}^BPA_{01}	6	1.000	1.000
**III-5-2**	NVA	12	BPA	6	NVA_{10}^BPA_{01}	6	0.707	0.500
