# Supplementary material for: Applying sequential pattern mining to investigate cerebrovascular health outpatients’ re-visit patterns
Source: PeerJ. 2018 Jul 9;6:e5183. doi: 10.7717/peerj.5183 (PMC6042480; doi:10.7717/peerj.5183)
Supplement: Appendix S1 [file peerj-06-5183-s003.pdf]

Appendix: List of Questions for Experts

Domain experts are expected to give their personal judgement to what extend they are agreed towards our findings, based on their knowledge and medical practice experiences through this questionnaire. All the questions present below are in accordance to our findings about outpatients' re-visit behavior with five level Likert scale, which range from 1 ("strongly disagree") to 5 ("strongly agree").

1. In general, younger people visit the clinic for medical check-ups more often than middle-age and elderly.

	1: strongly disagree		2: disagree		3: neutral		4: agree		5: strongly agree

2. Most of outpatients visit the clinic for medical check-ups several times because they have history of hypertension and/or diabetes.

	1: strongly disagree		2: disagree		3: neutral		4: agree		5: strongly agree

3. Most of middle-age outpatients have a high possibility of hypertension and/or obesity problem, but they are usually lack of concern for their health.

	1: strongly disagree		2: disagree		3: neutral		4: agree		5: strongly agree

4. Most of middle-age outpatients start concerning clinical visits for regular medical check-ups after they were diagnosed to have hypertension and/or diabetes.

	1: strongly disagree		2: disagree		3: neutral		4: agree		5: strongly agree

5. Most of elderly outpatients will begin to regularly seek medical check-ups after result of MRI and/or NV test shown abnormality.

	1: strongly disagree		2: disagree		3: neutral		4: agree		5: strongly agree

6. In general, outpatients start to do a regular visit for medical check-ups because they have been learning that abnormal blood pressure is the main reason that could lead to the increasing risk of stroke.

	1: strongly disagree		2: disagree		3: neutral		4: agree		5: strongly agree

7. Abnormal blood pressure typically influences outpatients' re-visit behavior when abnormal blood pressure is associated with obesity or high blood sugar level.

	1: strongly disagree		2: disagree		3: neutral		4: agree		5: strongly agree

8. In accordance to radiology diagnosis, the detection of abnormality shown by NV test is more likely to influence re-visit behavior of outpatients with abnormal blood pressure, rather than the result from MRI test.

	1: strongly disagree		2: disagree		3: neutral		4: agree		5: strongly agree

9. Radiology diagnosis, either MRI or NV test, plays an important role to the outpatients' re-visit behavior.

	1: strongly disagree		2: disagree		3: neutral		4: agree		5: strongly agree

10. Most outpatients who never get a radiology test, their re-visit behaviors are most likely influenced by any feature that leads to hypertension, associated with any features that leads to diabetes.

	1: strongly disagree		2: disagree		3: neutral		4: agree		5: strongly agree
